# Primary hyperparathyroidism secondary to parathyroid adenoma mimicking rickets - an unusual presentation

**DOI:** 10.11604/pamj.2023.45.136.40885

**Published:** 2023-07-20

**Authors:** Hardik Patel, Aditya Pundkar

**Affiliations:** 1 Department of Orthopedics, Datta Meghe Institute of Medical Sciences(DU), Sawangi, Wardha, Maharashtra, India

**Keywords:** Primary hyperparathyroidism, parathyroid adenoma, rickets

## Image in medicine

In rare cases, a parathyroid adenoma can mimic the signs and symptoms of rickets or contribute to ricket-like changes in the body. Parathyroid adenomas are benign tumors that develop in the parathyroid glands, which are responsible for regulating calcium and phosphate levels in the body. When an adenoma overproduces parathyroid hormone (PTH), it can disrupt the normal balance of calcium and phosphate, leading to ricket-like manifestations. Here are some ways in which a parathyroid adenoma can mimic rickets: 1) bone demineralization: excessive production of PTH by a parathyroid adenoma can cause increased bone resorption, resulting in bone demineralization. This can lead to weakened bones, similar to the under mineralization seen in rickets; 2) skeletal deformities: parathyroid adenomas causing hyperparathyroidism can lead to skeletal deformities, such as bowing of the long bones or other abnormalities, resembling the bone deformities seen in rickets; 3) growth delay: hyperparathyroidism resulting from a parathyroid adenoma can disrupt normal growth and development, causing growth delay in children; 4) vitamin D deficiency: in some cases, parathyroid adenomas can lead to vitamin D deficiency. Deficiency of vitamin D can contribute to ricket-like changes, as observed in rickets; 5) electrolyte imbalances: hyperparathyroidism caused by a parathyroid adenoma can disrupt the balance of calcium and phosphate in the body, leading to abnormal serum levels. These electrolyte imbalances can contribute to the development of ricket-like changes. If ricket-like changes are suspected, it is important to thoroughly evaluate the underlying cause. This typically involves conducting blood tests to assess calcium, phosphate, PTH, and vitamin D levels, as well as imaging studies to identify the presence of a parathyroid adenoma. Treatment of the adenoma usually involves surgical removal, which can help restore normal parathyroid function and resolve the ricket-like symptoms. Primary hyperparathyroidism (PHPT) is a rare disorder in pediatric age, with an estimated incidence of 2-5 cases in 100,000 live births. A 17-year-old male patient was brought to the orthopedics outpatient department (OPD) with complaints of joint pain and deformity for 2 years which were increasing over the period of time with no history of trauma. On examination, there were X-ray changes seen like mimicking rickets which were thinning and increased translucency of occipital bone of skull, rachitic rosary (beaded appearance of ribs) and cupping and flaring of the rib ends, physeal widening, epiphyseal cupping and fraying, metaphyseal fraying and splaying of distal radius and ulna and of the distal humerus and proximal radius and ulna and also looser zones (pseudo fractures), bowed or distorted long bones, delayed bone age seen.

**Figure 1:**
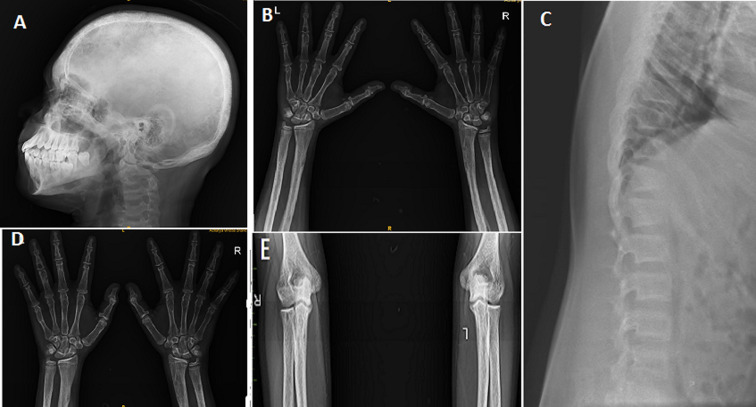
A) X-ray of thinning and increased translucency of the cranial bones, particularly in the occipital area of skull; B) X-ray of widened growth plate, metaphyseal fraying, splaying and cupping of the distal radius and ulna seen; C) X-ray of Rachitic rosary (beaded appearance on X-rays); D) X-ray showing cupping and fraying of the metaphysis of distal end of radius; E) X-ray of cupping and metaphyseal fraying and splaying of the distal humerus and proximal radius and ulna

